# Association of the Monocyte–to–High-Density Lipoprotein Cholesterol Ratio With Diabetic Retinopathy

**DOI:** 10.3389/fcvm.2021.707008

**Published:** 2021-09-21

**Authors:** Xixiang Tang, Ying Tan, Yi Yang, Mei Li, Xuemin He, Yan Lu, Guojun Shi, Yanhua Zhu, Yuanpeng Nie, Haicheng Li, Panwei Mu, Yanming Chen

**Affiliations:** ^1^Department of Endocrinology & Metabolism, The Third Affiliated Hospital of Sun Yat-sen University, Guangdong Provincial Key Laboratory of Diabetology, Guangzhou, China; ^2^VIP Medical Service Center, The Third Affiliated Hospital of Sun Yat-sen University, Guangzhou, China; ^3^Department of Clinical Immunology, The Third Affiliated Hospital of Sun Yat-sen University, Guangzhou, China

**Keywords:** monocyte to high-density lipoprotein cholesterol ratio, type 2 diabetes, diabetic retinopathy, biomarker, inflammation

## Abstract

**Background:** Chronic inflammation in type 2 diabetes mellitus (T2DM) is an essential contributor to the development of diabetic retinopathy (DR). The monocyte–to–high-density lipoprotein cholesterol (HDL-C) ratio (MHR) is a novel and simple measure related to inflammatory and oxidative stress status. However, little is known regarding the role of the MHR in evaluating the development of DR.

**Methods:** A total of 771 patients with T2DM and 607 healthy controls were enrolled in this cross-sectional study. MHR determination and eye examination were performed. The association of MHR with the prevalence of DR in T2DM patients was analyzed.

**Results:** The MHR in patients with DR was significantly higher than that in both non-DR diabetic patients (*P* < 0.05) and healthy controls (*P* < 0.01). No significance was observed in the MHR of different DR severity grades. Moreover, the MHR was similar between patients with non-macular oedema and those with macular oedema. Logistic regression analysis demonstrated that MHR was independently associated with the prevalence of DR in diabetic patients [odds ratio (OR) = 1.438, 95% confidence interval (CI): 1.249–1.655, *P* < 0.01]. After additional stratification by HbA1c level and diabetic duration, the MHR was still independently associated with the prevalence of DR.

**Conclusions:** Our study suggests that the MHR can be used as a marker to indicate the prevalence of DR in patients with T2DM.

## Introduction

Type 2 diabetes mellitus (T2DM) is a highly and rapidly evolving global health issue (1, 2), even in patients with prediabetes, the risk of macrovascular and microvascular disease were increased (3–5). Diabetic retinopathy (DR) is one of the most important diabetic microvascular complications and a leading cause of irreversible blindness among the working-age population around the world (6). Although the underlying molecular mechanisms of DR are not yet fully understood, abundant evidence indicates that inflammation plays a key role in the pathophysiology of DR (7). Various inflammatory cytokines and chemokines, such as ICAM-1, IL-1β, IL-6, IL-8, TNF-α, and MCP-1, have been reported to be elevated in the serum and vitreous and aqueous humor from diabetic patients with DR (8).

Monocytes are released from their precursors in bone marrow into the circulation and migrate to tissues and release proinflammatory cytokines at sites of inflammation, thereby affecting the severity of inflammation, which is considered an inflammatory biomarker (9). Additionally, plasma high-density lipoprotein cholesterol (HDL-C) has an inverse relationship with DR risk (10, 11). In addition, HDL-C has antioxidant efficacy to protect endothelial functions (12, 13). Therefore, the monocyte count–to–HDL-C ratio (MHR) can reflect the inflammatory status and is related to the development of disease associated with chronic inflammation. The MHR has been found to be associated with the occurrence and prognosis of cardiovascular diseases (CVDs), diabetic nephropathy and diabetic peripheral neuropathy (14–17). However, to the best of our knowledge, there are a few studies with small sample size that evaluated the associations of the MHR with DR and got contradictory conclusion (18, 19). Further studies investigating the relationship between MHR and DR in larger patient groups are needed. Thus, in the present study, we aimed to investigate the associations between the MHR and the prevalence of DR in adults with T2DM.

## Methods

### Study Population

A total of 1,378 subjects between the ages of 18 and 70 years, including 771 patients with T2DM and 607 healthy individuals, were included consecutively in this observational cross-sectional study between January 2016 and December 2018. T2DM patients were diagnosed based on the 1999 criteria of the World Health Organization (WHO) (20). The exclusion criteria were as follows: active or chronic inflammation, active infection, autoimmune diseases, hematological disorder, recent blood transfusion before enrolment, malignancy, acute or chronic renal/hepatic diseases, or coronary artery disease. Ethics approval was obtained from the Third Affiliated Hospital of Sun Yat-sen University Network Ethics Committee.

### Data Collection and Laboratory Analysis

Baseline information, including age, sex, comorbidities, smoking status, alcohol intake, medications, body height, weight, and blood pressure, was collected from medical records. Laboratory assessments consisted of fasting blood glucose, liver and renal function, uric acid, total cholesterol, triglycerides, low-density lipoprotein cholesterol (LDL-C), and high-density lipoprotein cholesterol (HDL-C), which were examined by a HITACHI (Tokyo, Japan) 7180 Automatic Analyser using 8-h overnight fasting blood samples. HbA1c was measured by high-performance liquid chromatography (HPLC) with a D-10 hemoglobin testing program (Bio-Rad). White blood cell measurement was performed with an automated hematology analyser XE-1200 (Sysmex, Kobe, Japan). The MHR ratio was calculated by dividing the monocyte count by HDL-C.

### Eye Examination

Eye examinations were performed on all participants according to standard operation procedures by trained ophthalmologists. The eye examinations included visual acuity measurements, tonometry, intraocular pressure, an anterior ocular structure, and fundus examination using a standard protocol. The external and anterior ocular segments were examined by slit lamp biomicroscopy (BQ900; Haag-Streit, Bern, Switzerland). Two 45° field digital, color, non-stereoscopic fundal photographs of each eye were taken in the macula-centered and posterior pole by a non-mydriatic auto-fundus camera (TRC-NW400 Non-Mydriatic Retinal Camera, Topcon, Tokyo, Japan).

### Assessment of DR

Two physicians made the assessment of DR independently (Kappa index = 0.919, *P* < 0.0001, indicating an excellent agreement between two physician). DR was diagnosed if any characteristic lesions existed as defined by the Early Treatment Diabetic Retinopathy Study (ETDRS). DR severity was further categorized into mild, moderate and severe non-proliferative DR (NPDR) and proliferative DR (PDR). Another important additional categorization in DR was diabetic macular oedema (DME) and non-DME (21).

### Statistical Analyses

Database management and statistical analysis were performed using PASW 22.0 for Windows (IBM Inc., Armonk, USA). Continuous variables are presented as the means ± standard deviation or median (interquartile range), while categorical variables are expressed as numbers (percentages). One-way ANOVA was applied for the comparison of continuous variables among groups, and a *post-hoc* test using Fisher's least significant difference (LSD) was used to determine which means differed following ANOVA. Differences in categorical variables were evaluated by Pearson's chi-square test. Univariate logistic regression analysis was performed to assess the non-adjusted relationships between 10^*^MHR and the prevalence of DR. Odds ratios (ORs) and 95% confidence intervals (CIs) were estimated for the association between DR and 10^*^MHR. Then, two multivariate logistic regression models were performed to adjust for confounding factors. Model 1 was adjusted for age, sex. Model 2 was additionally adjusted for body mass index, diabetes duration, smoking status, SBP, DBP, triglyceride, LDL-C, Cr, UA, FBG, HbA1c, and medications. To determine whether the duration of diabetes and glucose control status affect the relationship between MHR and the prevalence of DR, subgroup analyses were performed based on the duration of diabetes (<10 and ≥10 years) and HbA1c levels (<7.0 and ≥7.0%). A two-tailed *P* < 0.05 was considered statistically significant.

## Results

### The Clinical Characteristics of the Participants

Of 771 patients with T2DM, 164 (21%) were DR patients. The clinical characteristics of all participants are summarized in Table 1. No significant differences were observed in terms of age, sex, smoking status, or alcohol intake among all groups. Body mass index (BMI), blood pressure, monocyte counts, TGs, fasting blood glucose, HbA1c, and BUN in the healthy control group were lower than those in T2DM patients, while HDL-C and LDL-C were higher (all *P* < 0.05). Among all subjects with T2DM, the diabetic duration in the subjects with DR was much longer than that in the subjects with non-DR (*P* < 0.01). Higher neutrophil counts, monocyte counts, TGs, Cr, BUN, UA, and insulin use and lower levels of HDL-C were observed in patients with DR (all *P* < 0.05). However, no significant differences were observed in hyperlipidemia, blood pressure, fasting blood glucose, HbA1c, UACR, antiplatelet use, or statin use between subjects with DR and non-DR.

**Table 1:** Baseline characteristics.

**Variables**	**Health control** ** (*n* = 607)**	**NDR** ** (*n* = 607)**	**DR** ** (*n* = 164)**
Age, years	56.7 ± 8.6	57.1 ± 10.5	57.4 ± 9.8
Male, *n* (%)	286 (47.1)	294 (48.4)	93 (56.7)
BMI, kg/m2	23.0 ± 3.3	24.4 ± 3.4^**^	24.3 ± 3.8^**^
Diabetes duration, years	–	6.0 (2.0–11.0)	10.0 (5.0–15)^##^
Smoking, *n* (%)	140 (23.1)	143 (23.6)	47 (28.7)
Alcohol, *n* (%)	98 (16.1)	96 (15.9)	32 (19.5)
Hyperlipidemia, *n* (%)	–	38 (6.3)	8 (4.9)
Hypertension, *n* (%)	–	191 (31.5)	65 (39.6)
SBP, mmHg	117.4 ± 14.1	132.1 ± 18.7^**^	131.9 ± 18.6^**^
DBP, mmHg	75.1 ± 9.7	80.7 ± 11.0^**^	80.1 ± 10.9^**^
*Laboratory tests*			
White blood cells, 10^9^/L	6.22 ± 1.43	6.38 ± 1.61	6.99 ± 2.26
Neutrophils, 10^9^/L	3.62 ± 1.16	3.65 ± 1.21	4.28 ± 2.04^**^^#^
Lymphocytes, 10^9^/L	2.16 ± 0.55	2.11 ± 0.70	2.04 ± 0.59
Monocytes, 10^9^/L	0.43 ± 0.13	0.42 ± 0.12	0.47 ± 0.18^*^^#^
TC, mmol/L	5.00 ± 0.95	4.81 ± 1.20^*^	4.88 ± 1.49
TG, mmol/L	1.01 (0.75–1.47)	1.31 (0.92–1.87)^**^	1.32 (1.00–2.11)^**^^#^
HDL-C, mmol/L	1.30 ± 0.29	1.18 ± 0.28^**^	1.10 ± 0.28^**^^#^
LDL-C, mmol/L	3.34 ± 0.92	3.00 ± 1.01^**^	2.98 ± 1.05^**^
MHR, 10^9^/mmol	0.351 ± 0.147	0.370 ± 0.119^*^	0.458 ± 0.224^**^^##^
FBG, mmol/L	5.18 ± 0.68	9.51 ± 5.88^**^	9.92 ± 6.43^**^
HbA1c, %	5.3 ± 0.4	8.9 ± 2.5^**^	8.9 ± 2.3^**^
UACR, mg/g	–	0.99 (0.65–2.39)	1.25 (0.78–3.40)
Cr, umol/l	71.97 ± 15.55	71.32 ± 52.77	90.21 ± 52.05^**^^##^
BUN, umol/l	4.62 ± 1.13	5.71 ± 1.91^**^	6.35 ± 3.23^**^^##^
UA, umol/l	375.8 ± 101.9	352.7 ± 100.6^**^	375.3 ±106.1^#^
*Medications*			
Insulin, n (%)	–	189 (31.1)	75 (45.7)^#^
Metformin, *n* (%)	–	399 (65.7)	103 (62.8)
Glucosidase inhibitor, *n* (%)	–	185 (30.5)	52 (31.7)
Sulfonylureas, *n* (%)	–	169 (27.8)	55 (33.5)
DPP-4 inhibitors, *n* (%)	–	152 (25.0)	39 (23.8)
GLP-1R, *n* (%)	–	11 (1.8)	4 (2.4)
SGLT2 inhibitor, *n* (%)	–	13 (2.1)	4 (2.8)
Glinides, *n* (%)	–	19 (3.1)	5 (3.0)
Anti-platelet, *n* (%)	–	273 (45.0)	84 (51.2)
Statin, *n* (%)	–	431 (71.0)	112 (68.3)
ACEI/ARB, *n* (%)	–	151 (24.9)	49 (29.9)
β-blocker, *n* (%)	–	61 (10.0)	28 (17.1)^#^
CCB, *n* (%)	–	87 (14.3)	32 (19.5)
Diuretic, *n* (%)	–	10 (1.6)	6 (3.7)

*Data are mean (SD), median (25th to 75th percentile) or n (%).*

*BMI, body mass index; SBP, systolic blood pressure; DBP, diastolic blood pressure; FBG, Fasting blood glucose; UACR, urine albumin to creatinine rate; Cr, plasma creatinine; BUN, blood urea nitrogen; UA, uric acid; TC, total cholesterol; TG, triglycerides; HDL-C, high density lipoprotein cholesterol; LDL-C, low density lipoprotein cholesterol; ACEI/ARB, angiotensin converting enzyme inhibitor/angiotensin receptor blocker; CCB, calcium channel blockers.*

* *P < 0.05 vs. Health control,*

** *P < 0.01 vs. Health control,*

# *P < 0.05 vs. NDR,*

## *P < 0.01 vs. NDR*.

### The Association Between MHR and DR

**Figure 1:**
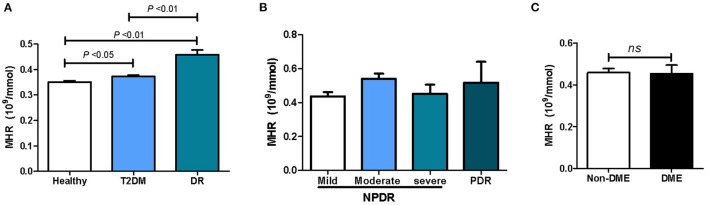
The association between the MHR and patients with DR. **(A)** MHR levels in healthy controls, diabetic patients with and without DR; **(B)** MHR levels in DR subjects with different severity; **(C)** MHR levels between subjects with DME and non-DME.

Compared to that in the healthy controls, the level of MHR was remarkably increased in patients with T2DM (*P* < 0.01), as shown in Figure 1A. The MHR in patients with DR was significantly higher than that of both the diabetic patients without DR and the healthy controls (*P* < 0.01). No significant difference was observed in DR subjects with different severity or between subjects with DME and non-DME subjects (Figures 1B,C).

### Univariate and Multivariate Logistic Regression Analysis

Univariate logistic regression analysis demonstrated that 10^*^MHR was associated with the development of DR [OR (95% CI) = 1.435 (1.277–1.614), *P* < 0.001] (Table 2). After adjusting for age and sex, 10^*^MHR was still independently related to the development of DR [OR (95% CI) = 1.424 (1.263–1.605), *P* < 0.001]. When further adjusting for BMI, diabetes duration, smoking status, SBP, DBP, triglyceride, LDL-C, Cr, UA, FBG, HbA1c, and medications, 10^*^MHR remained independently associated with the prevalence of DR [OR (95% CI) = 1.438 (1.249–1.655), *P* < 0.001].

**Table 2:** Logistic regression analysis assessing the association of the MHR with diabetic retinopathy.

**Variables**	**Univariate**			Model 1^*^			Model 2^*^		
	**OR**	**95%CI**	***P*-value**	**OR**	**95%CI**	***P*-value**	**OR**	**95%CI**	***P*-value**
10^*^MHR, 10^8^/mmol									
Overall	1.435	1.277–1.614	<0.001	1.424	1.263–1.605	<0.001	1.438	1.249–1.655	<0.001
*Stratified by HbA1c*									
<7.0	1.440	1.156–1.793	0.001	1.519	1.195–1.931	0.001	1.739	1.232–2.455	0.002
≥7.0	1.433	1.248–1.646	<0.001	1.411	1.226–1.623	<0.001	1.432	1.206–1.699	<0.001
*Stratified by diabetic duration*									
<10	1.375	1.168–1.618	<0.001	1.373	1.157–1.628	<0.001	1.304	1.070–1.589	0.008
≥10	1.500	1.263–1.781	<0.001	1.465	1.233–1.740	<0.001	1.724	1.354–2.195	<0.001

* *Model 1 was adjusted for age, sex.*

*Multivariate regression analysis model 2 was adjusted for age, sex, BMI, diabetes duration, smoking status, SBP, DBP, triglyceride, LDL-C, Cr, UA, FBG, HbA1c, and medications*.

### Subgroup Analysis

The most consistent risk factors for the development of DR are long duration of diabetes and hyperglycemia (6, 22, 23). A reasonable HbA1c level is below or around 7% and longer duration of diabetes was 8–11 years according to the American Diabetes Association (ADA) and the European Association for the Study of Diabetes (EASD) (24, 25). To preclude the influence of the duration of diabetes and glucose control status, which were introduced in the subgroup analysis, as shown in Table 2, we further performed a subgroup regression analysis stratified by HbA1c levels (<7 vs. ≥7%) and the duration of diabetes (<10 vs. ≥10 years). In multivariate logistic regression Model 2, the 10^*^MHR group had a significantly higher prevalence of DR regardless of the level of HbA1c (*P* < 0.05) and diabetic duration (*P* < 0.01).

## Discussion

In the present study, the results provide evidence about the unique association between the MHR and DR. Elevated MHR levels were associated with increased odds of DR, independent of a variety of conventional DR risk factors. However, we did not detect a significant association between the MHR level and DR severity or macular edema in patients with T2DM.

Inflammation markers and monocytes play an important role in the development of diabetic complications (16, 26, 27). Retinal chronic inflammation plays a pivotal role in the development of DR (6, 7). The levels of monocytes are increased in the retinal vessels and differentiate into macrophages that secrete inflammatory cytokines and growth factors adhering to the outer surface of retinal capillaries, leading to the breakdown of the blood retinal barrier, increased retinal vascular permeability and capillary non-perfusion, which are considered characteristic pathologic features in early DR (28, 29). In the present study, neutrophil and monocyte counts were remarkably increased in patients with DR compared to healthy controls or patients without DR, which is consistent with the findings in previous studies. Hyperglycemia enhances the inflammatory status to release more neutrophils and monocytes from bone marrow and then recruit them into the retinal vessels, causing damage to these vessels (30).

Lipid disorders seem to contribute to the development and progression of DR (31, 32). Accumulated evidence indicates that poor control of triglycerides and LDL is associated with the incidence and progression of DR, while higher HDL-C levels and the use of lipid-lowering medication significantly reduce the risk of DR (33–35). Our results also showed higher levels of triglycerides and lower HDL in DR individuals.

Recently, the MHR has emerged as a novel and convenient marker with the integration of proinflammatory and anti-inflammatory factors (14–19). Emerging data suggest that higher MHR values are associated with various diseases or organ dysfunctions, such as endothelial dysfunction in Behçet disease, the presence and severity of metabolic syndrome, polycystic ovary syndrome, cardiac syndrome X, serum albumin level saphenous vein graft disease in coronary bypass, the high SYNTAX score in patients with stable coronary artery disease, asymptomatic organ damage in patients with primary hypertension, left atrial remodeling in atrial fibrillation, abdominal aortic aneurysm size, myocardial infarction, and CVD in patients with obstructive sleep apnea syndrome (14, 15, 36–43). It has also been shown that MHR is an independent predictor of in-hospital and long-term mortality and major adverse cardiac events in patients with acute coronary syndromes or a post-PCI status (44). Therefore, the MHR is a new prognostic marker in several CVDs, which are associated with inflammation. In addition, accumulated evidence has shown that the MHR is related to diabetes and diabetic complications (16, 45) and ocular disorders, including pseudoexfoliation syndrome (46), glaucoma, branch retinal vein occlusion (47), and central serous retinopathy (48). MHR values are increased in patients with diabetes compared to healthy controls, and an elevated MHR can predict diabetic nephropathy and diabetic axonal polyneuropathy (49). Moreover, there are a few studies with small sample size that evaluated the associations of the MHR with DR and got contradictory conclusions. Işil Çakir et al.' study showed that MHR was significantly higher in DR group than T2DM without DR group and found that specificity and sensitivity of MHR in detecting DR were relatively low (18); while it can be inferred that MHR is not affected by diabetes, but only by the proliferation process in Inhsan Solmaz et al.' study (19). Then, in the present study with larger sample size showed that diabetes, a chronic inflammatory disease, yields an elevated MHR level, and an elevated MHR can be an useful biomarker for DR independent of conventional risk factors, but not predict the severity stage of DR, which is not consistent with the previous study (18, 19). To date, several studies in addition to our study have consistently demonstrated that the MHR is a reliable factor for inflammation and is associated with diabetic micro- and macrovascular complications.

Furthermore, compared to other expensive inflammatory markers, such as interleukin factor (IL)-1, IL-6, tumor necrosis factor-α, and monocyte chemo-attractant protein-1, the MHR can be easily calculated from a simple blood analysis, making the use of this index more practical, cost-effective, and useful to predict DR (8).

It is important to note the limitations of our investigation. First, it was a single-center cross-sectional study; thus, causal relationships between MHR and DR cannot be confirmed. These findings should be cautiously interpreted, and further prospective studies are needed. Second, we did not exclude subjects with macrovascular complications, such as coronary artery disease, because the percentage of subjects with these complications did not vary significantly between the DR and non-DR groups. Finally, additional inflammation markers, such as CRP, interleukin factor (IL)-6, and tumor necrosis factor-α, were not evaluated herein.

In summary, the present study suggests that elevated MHR is a convenient and effective measurement for predicting the presence of DR in patients with T2DM.

## Data Availability Statement

The raw data supporting the conclusions of this article will be made available by the authors, without undue reservation.

## Ethics Statement

The studies involving human participants were reviewed and approved by Ethics approval was obtained from the Third Affiliated Hospital of Sun Yat-sen University Network Ethics Committee. Written informed consent for participation was not required for this study in accordance with the national legislation and the institutional requirements.

## Author Contributions

XT, YT, and YC contributed to the study design, formal analysis, and writing—original draft. XT, YT, YY, XH, ML, and HL contributed to the data acquisition and curation. YZ and YN contributed to the literature research. YL, GS, and PM revised the manuscript. All authors contributed to the article and approved the submitted version.

## Funding

This study was funded by National Key R&D Program of China (2017YFA0105803), the National Natural Science Foundation of China (81770826, 82000278), the 5010 Clinical Research Projects of Sun Yat-sen University (2015015), the Key Area R&D Program of Guangdong Province (2019B020227003), the Science and Technology Plan Project of Guangzhou City (202007040003), the Guangdong Basic and Applied Basic Research Foundation (2020A1515010599), and the fostering special funding projects of the National Natural Science Foundation of China in the third affiliated hospital of SYSU (2020GZRPYQN04).

## Conflict of Interest

The authors declare that the research was conducted in the absence of any commercial or financial relationships that could be construed as a potential conflict of interest.

## Publisher's Note

All claims expressed in this article are solely those of the authors and do not necessarily represent those of their affiliated organizations, or those of the publisher, the editors and the reviewers. Any product that may be evaluated in this article, or claim that may be made by its manufacturer, is not guaranteed or endorsed by the publisher.
